# Prevalence of bullying and victimization among children in early elementary school: Do family and school neighbourhood socioeconomic status matter?

**DOI:** 10.1186/1471-2458-12-494

**Published:** 2012-07-02

**Authors:** Pauline W Jansen, Marina Verlinden, Anke Dommisse-van Berkel, Cathelijne Mieloo, Jan van der Ende, René Veenstra, Frank C Verhulst, Wilma Jansen, Henning Tiemeier

**Affiliations:** 1 Department of Child and Adolescent Psychiatry/Psychology, Erasmus MC-University Medical Center, PO-BOX 2060, 3000, CB, Rotterdam, the Netherlands; 2 The Generation R Study Group, Erasmus MC-University Medical Center, Rotterdam, the Netherlands; 3 Municipal Public Health Service Rotterdam Rijnmond, Rotterdam, the Netherlands; 4 Department of Public Health, Erasmus MC-University Medical Center, Rotterdam, the Netherlands; 5 Department of Sociology, University of Groningen, Groningen, the Netherlands; 6 Department of Psychology, University of Turku, Turku, Finland; 7 Department of Epidemiology, Erasmus MC-University Medical Center, Rotterdam, the Netherlands

**Keywords:** Bullying, Victimization, Socioeconomic status, Children

## Abstract

**Background:**

Bullying and victimization are widespread phenomena in childhood and can have a serious impact on well-being. Children from families with a low socioeconomic background have an increased risk of this behaviour, but it is unknown whether socioeconomic status (SES) of school neighbourhoods is also related to bullying behaviour. Furthermore, as previous bullying research mainly focused on older children and adolescents, it remains unclear to what extent bullying and victimization affects the lives of younger children. The aim of this study is to examine the prevalence and socioeconomic disparities in bullying behaviour among young elementary school children.

**Methods:**

The study was part of a population-based survey in the Netherlands. Teacher reports of bullying behaviour and indicators of SES of families and schools were available for 6379 children aged 5–6 years.

**Results:**

One-third of the children were involved in bullying, most of them as bullies (17%) or bully-victims (13%), and less as pure victims (4%). All indicators of low family SES and poor school neighbourhood SES were associated with an increased risk of being a bully or bully-victim. Parental educational level was the only indicator of SES related with victimization. The influence of school neighbourhood SES on bullying attenuated to statistical non-significance once adjusted for family SES.

**Conclusions:**

Bullying and victimization are already common problems in early elementary school. Children from socioeconomically disadvantaged families, rather than children visiting schools in disadvantaged neighbourhoods, have a particularly high risk of involvement in bullying. These findings suggest the need of timely bullying preventions and interventions that should have a special focus on children of families with a low socioeconomic background. Future studies are necessary to evaluate the effectiveness of such programs.

## Background

Bullying and victimization are widespread phenomena in childhood and can take several forms, such as name calling, gossiping, exclusion, and hitting or pushing [1]. Children’s involvement in bullying, either as a bully or victim, has a serious impact on their well-being [2-8]. Victims are at increased risk of future poor physical health, low self-esteem, and psychiatric problems, such as anxiety disorders, depression, and psychotic symptoms. Bullies have more behavioural problems and a poorer emotional adjustment later in life. Moreover, victims and bullies tend to perform less well at school than children who are not involved in bullying [3,6]. Children can also be involved in bullying behaviour both as bully *and* as victim, and these so-called bully-victims have a particularly high risk of later psychosocial problems [9,10]. These adverse consequences are independent of pre-existing behavioural and emotional problems at the time the bullying and victimization takes place [2-8].

Several prevalence studies indicated that bullying and victimization are a common problem in elementary and secondary school classes [3-8,11-14]. Large cross-national research, for instance, showed that on average 27% of children in secondary schools were involved in bullying: approximately 13% of the children reported being a victim of bullying, 11% a bully, and 4% a bully-victim [14]. In general, boys are more often involved in bullying than girls [12-15]. In contrast to the abundance of large-scale studies in children in secondary school and higher grades of elementary school, there is little evidence that bullying and victimization already exists among younger children [16-20]. A few small-scale studies in kindergarten and the first grades of elementary school focused only on victims and reported varying prevalence rates of victimization ranging from 2% to 27% [16,17,19]. Hence, it remains rather unclear to what extent bullying and victimization affects the lives of young children [16-20].

It is important that children with an increased risk of becoming a bully or victim are identified at a young age so as to facilitate timely prevention of bullying and victimization. Identification is enhanced by knowledge on determinants and predictors of bullying behaviour. Previously, studies on determinants of bullying mainly focused on individual traits of children and on the influence of parenting styles [6,21,22]. For instance, bullies often have an impulsive and dominant temperament and are frequently exposed to harsh child-rearing practices at home. Recently, considerable attention has been paid to socioeconomic predictors of school bullying. This has led to the postulation that involvement in bullying behaviour might explain part of the socioeconomic disparities in mental health problems [23]. For instance, it has been shown that adolescents from families with a lower socioeconomic status (SES) are more often victimized and face more severe long-term mental health consequences of this victimization as compared to victims from more affluent social backgrounds [23]. Other studies have confirmed that victimization rates were higher among children with a low socioeconomic background as indicated by their parents’ low-skill occupations or low educational attainment, lack of material resources, and single parenthood [19,24-28]. Like victimization, bullying seems to be socially patterned by parental socioeconomic status as well [13,28,29]. Besides family SES, school neighbourhood SES might also predict bullying behaviour because characteristics of school neighbourhoods, e.g. crime rates, social support and control, and common norms and values, are likely to influence children’s behaviour [30,31].

The aim of this study is to assess the prevalence of bullying and victimization among young elementary school children and to examine socioeconomic disparities in bullying behaviour. We hypothesize that school neighbourhood SES is associated with bullying behaviour independent of family SES. To improve understanding of bullying, three types of involvement in bullying are studied: victims, bullies, and bully-victims. The present study is embedded in a large population-based sample of 5- and 6-year old children in the second grade of elementary school. Teacher reports of bullying are used as teachers can observe peer interactions during daily school curriculum and, arguably, provide more objective information on bullying behaviour than parents [32].

## Methods

### Design

Data from the population-based Rotterdam Youth Health Monitor of the Municipal Public Health Service were used. This health surveillance system is part of government approved routine health examinations and monitors the health and well-being of children and youth living in Rotterdam and surrounding areas. The information is used for individual referral and guides youth policies of schools, neighbourhoods, and the municipality. The Medical Ethical Committee EUR/AZR of the Erasmus University/Academic Hospitals approved the use of data obtained by the Municipal Public Health Service for routine monitoring purposes for scientific publications (MEC 168.344/1998/43). The present study is based on data obtained from parental and teacher questionnaires. Parents were informed about the teacher questionnaire and were free to withdraw consent. Active consent is not required by Dutch law.

### Study population

For the present study, we used 2008/2009 survey data of children aged 5–6 years (n = 11,419). The elementary school teachers of these children were asked to complete a questionnaire for each child in their class. This resulted in teacher reports of bullying behaviour for 8871 children (response rate 77.7%). Parental questionnaires containing information about indicators of SES were available for 6376 of these children.

### Measures

#### Bullying and victimization

Bullying and victimization during the past three months were studied as outcome. The teacher of each elementary school child rated the occurrence of four victimization and four bullying items [20]. The victimization items assessed 1) “whether a child was physically victimized by other children, for instance by being hit, kicked, pinched, or bitten” (further referred to as physical victimization); 2) “whether a child was verbally victimized, such as being teased, laughed at, or called names” (verbal victimization); 3) “whether a child was excluded by other children” (relational victimization); and 4) “whether belongings of a child were hidden or broken” (material victimization). Bullying was assessed with the perpetration form of these four items, e.g. “Whether a child physically bullied other children”. Examples of physical and verbal victimization/bullying were added to the items, and we provided concrete descriptions of relational and material victimization/bulling. A pilot study had indicated that teachers thought these examples and descriptions were more helpful for consistent answering of the items than a formal definition of bullying. Each item was rated on a four-point rating scale ranging from “Never or less than once per month” to “More than twice per week”. Children with a “Never or less than once per month”-rating on all four bullying and four victimization items were classified as uninvolved children. Children were classified as victims if they experienced any of the four victimization types at least once a month. Likewise, children were classified as bullies if they perpetrated any of the forms of bullying at least once a month. Children meeting the criteria of both bullies *and* victims were categorized as bully-victims.

#### Family socioeconomic status

Information on indicators of family socioeconomic status was assessed by a parental questionnaire and, thus, obtained independently from the teacher questionnaire. The educational level of both parents was considered as an indicator of family SES because education structures income and occupation (economic status), but also reflects non-economic social characteristics, such as general knowledge, problem-solving skills, literacy, and prestige [33,34]. The highest attained educational level of mothers and fathers was divided into: “Primary education”, which typically corresponds to ≤8 years of education; “Lower vocational training”, corresponding to 9–12 years of education; “Intermediate vocational training”, equivalent to 13–15 years of education; “Higher vocational training”, which corresponds to 16–17 years of education; and “Higher academic education”, equivalent to 18 years of education or more [35]. Given that the highest obtained schooling significantly structures occupational levels [33], we included (un)employment status − instead of occupational level − as an indicator of family SES. Unemployment is generally seen as a strong indicator of low socioeconomic status [34]. Employment status was categorized as “At least one of the parents employed” and “Both parents unemployed”. The latter category indicated that none of the parents had paid employment and were comprised of parents who were in the categories of housewife/husband, student, job-seeker, or social security or disability benefit recipient. Proxy indicators of low SES used in this study were a young parental age and single parenthood, which was defined as “parents not living together”.

#### School neighbourhood socioeconomic status

The *SES of school neighbourhoods* was determined by linking the school postal code areas with neighbourhood level status scores obtained from the Netherlands Social and Cultural Planning Office [36]. These status scores are based on educational levels, income, and unemployment rates in neighbourhoods between 2002 and 2006. The status scores reflect standard deviation scores from a nation wide mean of zero and range between −5.5 and 3.3. The mean status score in the study area was −0.41 (100% range: -3.8 to 3.3). Lower scores indicate more social disadvantage. The SES scores of school neighbourhoods were divided into quartiles.

#### Confounders and multilevel measures

Child gender, age and national origin were considered as possible confounding factors in the association between SES and bullying behaviour. The national origin of the child was based on country of birth of both parents, as assessed by the parental questionnaire. A child was classified as non-Dutch if one or both parents were born abroad [37].

### Statistical analyses

The distribution of separate bullying and victimization items was analyzed, stratified by child gender. Differences in prevalence of bullying and victimization items were also presented by educational level of the mother, as maternal education is considered to be one of the strongest socioeconomic markers of child health and behaviour [38]. Differences by gender and by maternal educational level were tested with the *χ*^*2*^-statistic. Based on the separate bullying and victimization items, children were categorized as uninvolved children, victims, bullies, or bully-victims. The relation between SES indicators and involvement in bullying and victimization was examined with multinomial logistic regression analyses. We calculated the odds ratios (ORs) for each of the three categories of involvement in bullying (victim, bully, bully-victim) as compared to uninvolved children (reference group). The association of SES indicators with involvement in bullying and victimization was examined first for each indicator separately. These analyses were adjusted for confounding variables child age, gender, and national origin. Next, to estimate whether family SES and school neighbourhood SES independently contributed to the risk of bullying behaviour, we performed regression analyses including indicators of family SES and school neighbourhood SES in one model. As maternal and paternal education (Spearman’s rho = 0.63) and age of mothers and fathers (Pearson’s r = 0.59) were highly correlated, only maternal education and age were included in the full model. The model was then repeated, including paternal education and age instead of maternal education and age. The effect estimates of the full model include the maternal variables. To obtain a p-value for trend, the analyses were repeated, this time including educational level and school neighbourhood SES as continuous variables. Data was analyzed in a two-level structure with children clustered within classes because teachers rated bullying and victimization for all children in their class. All variables were analyzed at the individual level, except for school neighbourhood SES, which was included as a class-level variable. In the multivariate analyses, missing values on the SES variables and confounders were dealt with by the full information maximum likelihood (FIML) method in Mplus Version 5 [39]. FIML estimates model parameters and standard errors using all available data while adjusting for the uncertainty associated with missing data [40]. Analyses were performed using SPSS Version 17.0 [41] and Mplus.

### Non-response analysis

The distribution of involvement in bullying and victimization was compared between children with (n = 6379) and without (n = 2492) the parental questionnaire available. Children with missing data were more often involved in bullying than children without missing data (42.0% vs. 33.9%, p < 0.001). This was reflected in higher percentages of victims (5.4% vs. 4.0%), bullies (18.2% vs. 16.9%), and bully-victims (18.4% vs. 13.1%).

## Results

The study population was composed of 51% boys. More than half of the children had a Dutch background (57%). Most parents had an intermediate vocational training (mothers: 36%; fathers: 32%), which typically corresponds to 13 to 15 years of education. In 13% of the families, neither of the parents had paid employment.

The frequency of various bullying and victimization items is presented in Table 1. Physical bullying (16%), verbal bullying (22%), and relational bullying (27%) were highly common behaviors in early elementary school. Likewise, physical victimization (8%), verbal victimization (11%), and relational victimization (9%) were also common, although to a slightly lesser degree. Physical, verbal, and material victimization and bullying occurred more often in boys than in girls, while relational victimization and bullying was more prevalent among girls. A rather small percentage of bullying and victimization occurred on a weekly basis, e.g. physical victimization 1%. Additional file 1: Table S1 shows a clear socioeconomic gradient (as indicated by the level of education of the mother) for the types of bullying and victimization: physical, verbal, relational and material bullying, and victimization were all more prevalent among children of mothers with a low educational level as compared to children of higher educated mothers.

**Table 1:** Prevalence of victimization and bullying for all children and by gender

		**Percentage based on past 3 months**		
Items		Never^#^	Monthly	Weekly^§^
Victimization				
Physical	All	91.7	7.1	1.2
	Boys	88.0	9.9 ^a^	2.1 ^b^
	Girls	95.6	4.2	0.2
Verbal	All	89.4	9.2	1.4
	Boys	87.5	10.8 ^a^	1.7 ^b^
	Girls	91.5	7.4	1.1
Relational	All	91.1	7.6	1.3
	Boys	91.6	6.8 ^a^	1.6 ^b^
	Girls	90.7	8.4	0.9
Material	All	99.3	0.7	0.1
	Boys	99.0	0.9 ^a^	0.1
	Girls	99.5	0.5	0
Bullying				
Physical	All	84.1	11.3	4.6
	Boys	76.6	16.1 ^a^	7.3 ^b^
	Girls	92.0	6.3	1.7
Verbal	All	77.9	16.8	5.3
	Boys	73.2	19.4 ^a^	7.4 ^b^
	Girls	82.9	14.0	3.1
Relational	All	83.4	13.8	2.8
	Boys	85.6	11.4 ^a^	2.9
	Girls	81.1	16.3	2.6
Material	All	97.1	2.4	0.5
	Boys	96.0	3.2 * ^a^	0.9 ^b^
	Girls	98.3	1.5	0.2

Notes Table 1: ^#^ Never or less than once per month.

^§^ The categories of “One to two times per week” and “More than twice per week” were collapsed into the category “Weekly” due to very low prevalences.

^a^ Prevalence of never vs. monthly or ^b^ vs. weekly involvement in bullying differs significantly between boys and girls, p < 0.05.

**Figure 1:**
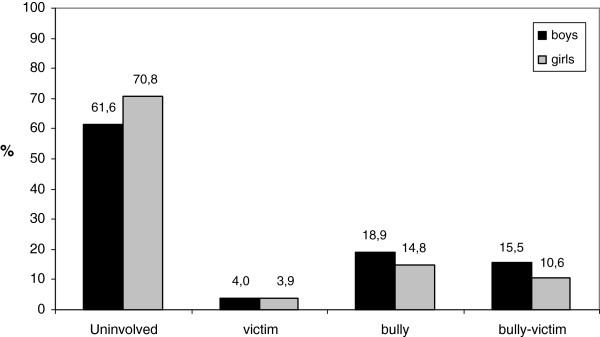
Prevalence of involvement in bullying and victimization by gender (n = 6376).

Based on the eight bullying and victimization items presented in Table 1, children were classified in four groups: uninvolved children, victims, bullies, and bully-victims. Figure 1 shows the distribution of these groups stratified by gender. The majority of children in early elementary school (66.1%, n = 4214) were not involved in bullying and victimization. Among those children involved, 4.0% was a victim of bullying (n = 252), 16.9% a bully (n = 1075), and 13.1% a bully-victim (n = 835). Boys were more often bullies (p < 0.001) or bully-victims (p < 0.001) than girls were.

Table 2 shows the association between SES and risk of involvement in bullying and victimization. Indicators of family SES were highly associated with bully and bully-victim status: single parenthood, young parental age, low educational level of parents, and parental unemployment increased the risk of children being a bully or bully-victim (see Table 2). Of all indicators of family SES, only low educational level of parents was associated with victimization (p-values for trend = 0.01 and 0.02 for maternal and paternal education, respectively). The relationship of school neighbourhood SES with bullying and victimization is also presented in Table 2. Low school neighbourhood SES increased the risk of being a bully or bully-victim although the latter was only marginally significant (low SES: OR = 1.45, 95% CI: 1.00-2.10).

**Table 2:** Effects of socioeconomic determinants on involvement in bullying

		**Odds ratios for involvement in bullying and victimization (95%-CI)**^#^			
		**Basic model without mutual adjustment for indicators of SES**			
**Indicators of socioeconomic status**	**N**^§^	**Uninvolved (n = 4214)**	**Victim (n = 252)**	**Bully (n = 1075)**	**Bully-victim (n = 835)**
Age mother (per 5 year decrease)	6161	Reference	1.09 (0.96-1.24)	1.06 (1.00-1.15)	1.15 (1.06-1.25)
Age father (per 5 year decrease)	6161	Reference	1.05 (0.94-1.17)	1.06 (1.00-1.12)	1.15 (1.07-1.23)
Single parenthood	6155	Reference	1.17 (0.80-1.72)	1.52 (1.14-1.80)	1.35 (1.05-1.74)
Educational level mother: Higher academic	655	Reference	Reference	Reference	Reference
Higher vocational	1020		1.36 (0.71-2.60)	1.06 (0.77-1.60)	1.32 (0.86-2.02)
Intermediate vocational	1952		1.40 (0.76-2.56)	1.20 (0.90-1.75)	1.56 (1.05-2.33)
Lower vocational	1318		1.70 (0.91-3.18)	1.30 (0.96-1.94)	1.99 (1.31-3.01)
Primary education	414		2.23 (1.08-4.64)	1.35 (0.92-2.24)	2.21 (1.33-3.66)
*p-value for trend*			*0.02*	*0.01*	*0.001*
Educational level father: Higher academic	655	Reference	Reference	Reference	Reference
Higher vocational	1020		1.06 (0.58-1.94)	1.07 (0.80-1.43)	1.14 (0.77-1.69)
Intermediate vocational	1952		1.37 (0.80-2.35)	1.06 (0.81-1.39)	1.14 (0.79-1.65)
Lower vocational	1318		1.81 (1.04-3.13)	1.29 (0.98-1.70)	1.54 (1.05-2.25)
Primary education	414		1.80 (0.87-3.74)	1.68 (1.16-2.45)	2.00 (1.22-3.25)
*p-value for trend*			*0.01*	*0.002*	*0.008*
Employment: At least one parent employed	4852	Reference	Reference	Reference	Reference
Both parents unemployed	745		1.00 (0.62-1.60)	1.15 (0.89-1.49)	1.22 (0.90-1.66)
School neighbourhood SES: High	1861	Reference	Reference	Reference	Reference
Mid-high	1678		0.81 (0.58-1.14)	1.00 (0.79-1.28)	0.93 (0.66-1.30)
Mid-low	1524		1.14 (0.85-1.51)	1.10 (0.87-1.40)	0.95 (0.67-1.33)
Low	1313		0.84 (0.61-1.15)	1.13 (0.90-1.43)	1.07 (0.74-1.54)
*p-value for trend*			*0.17*	*0.55*	*0.18*

Footnotes Table 2: ^#^ Analyses adjusted for child gender, age, and national origin. ^§^ N varies due to missing data in the SES indicators, total n = 6376.

Finally, the independent effect of family SES and school neighbourhood SES on risk of involvement in bullying behaviour was estimated. Table 3 shows that, adjusted for family SES, the association between school neighbourhood SES and involvement in bullying was not significant anymore. The ORs for the family SES variables were attenuated slightly, but all except parental employment status remained significant predictors of bully or bully-victim status. Again, victimization was only predicted by parental education. Results were approximately the same if paternal age and education were included in this model instead of maternal age and education.

**Table 3:** Effects of socioeconomic determinants on involvement in bullying with mutual adjustment for other socioeconomic determinants

		**Fully adjusted odds ratios for involvement in bullying and victimization (95%-CI)**^#^			
		**Model with mutual adjustment for indicators of SES**			
**Indicators of socioeconomic status**	**N**	**Uninvolved (n = 4214)**	**Victim (n = 252)**	**Bully (n = 1075)**	**Bully-victim (n = 835)**
Age mother (per 5 year decrease)	6161	Reference	1.12 (0.99-1.26)	1.10 (1.03-1.18)	1.18 (1.08-1.28)
Age father (per 5 year decrease)	5825	Reference	1.07 (0.96-1.19)	1.07 (1.01-1.13)	1.15 (1.07-1.24)
Single parenthood	6155	Reference	1.23 (0.89-1.70)	1.69 (1.41-2.02)	1.58 (1.27-1.95)
Educational level mother: Higher academic	655	Reference	Reference	Reference	Reference
Higher vocational	1020		1.20 (0.60-2.38)	1.12 (0.81-1.54)	1.33 (0.86-2.04)
Intermediate vocational	1952		1.22 (0.65-2.32)	1.38 (1.03-1.85)	1.60 (1.07-2.40)
Lower vocational	1318		1.48 (0.76-2.88)	1.51 (1.11-2.06)	1.98 (1.29-3.02)
Primary education	414		2.05 (0.97-5.25)	1.56 (1.06-2.31)	2.18 (1.31-3.63)
*p-value for trend*			*0.02*	*0.01*	*0.001*
Educational level father: Higher academic	837	Reference	Reference	Reference	Reference
Higher vocational	963		1.11 (0.60-2.06)	1.08 (0.79-1.47)	1.15 (0.78-1.69)
Intermediate vocational	1613		1.45 (0.83-2.53)	1.12 (0.84-1.49)	1.13 (0.79-1.62)
Lower vocational	1250		1.85 (1.05-3.25)	1.41 (1.05-1.90)	1.51 (1.03-2.19)
Primary education	352		1.82 (0.87-3.79)	1.90 (1.30-2.79)	1.99 (1.23-3.20)
*p-value for trend*			*0.01*	*0.002*	*0.008*
Employment: At least one parent employed	4852	Reference	Reference	Reference	Reference
Both parents unemployed	745		1.17 (0.78-1.74)	1.61 (1.29-2.00)	1.57 (1.21-2.04)
School neighbourhood SES: High	1861	Reference	Reference	Reference	Reference
Mid-high	1678		0.77 (0.56-1.07)	1.24 (0.96-1.59)	1.10 (0.77-1.57)
Mid-low	1524		0.98 (0.67-1.44)	1.29 (1.00-1.67)	1.14 (0.79-1.64)
Low	1313		0.86 (0.59-1.24)	1.49 (1.14-1.93)	1.45 (1.00-2.10)
*p-value for trend*			*0.17*	*0.55*	*0.18*

Footnotes Table 3: ^#^Analyses include child gender, age, national origin, and all SES-indicators, except age and education of fathers. ORs of paternal variables are derived by repeating the analysis including paternal and excluding maternal age and education.

## Discussion

This study showed significant socioeconomic disparities in bullying and victimization in early elementary school: children of lower socioeconomic families had a higher risk of being involved in bullying - either as victim, bully, or bully-victim - than children with a higher socioeconomic background. Before these socioeconomic disparities can be discussed, it is important to consider the reported prevalence rates first. Our findings suggest that bullying and victimization are relatively common problems in the lowest grades of elementary school with about one third of the children being involved. More specifically, we showed that 4% of the children were victims, whereas many children were involved as bullies (17%) or bully-victims (13%). These prevalence estimates, particularly of bullies and bully-victims, are somewhat higher than previously reported prevalence rates among older children and adolescents in the Netherlands and in other countries [14]. However, bullying behaviour tends to decline with age [14,42]. Possibly, young children solve peer problems with bully behaviour while children’s experiences, increasing assertiveness, and changes in capabilities and social skills might result in more adequate problem solving skills at older ages [43]. Our finding that bully-victims are highly represented while pure victimship is much less common contrasts with previous research among older children indicating that bully-victims are relatively rare compared with pure victims. It might be that children shift between categories such that young bully-victims become pure victims over time; however, this hypothesis and the possible explanations for such a shift can only be examined in a study with a longitudinal design. Yet, the high prevalence of children classified as bully-victims at this young age might also reflect general conflicts between children rather than bullying behaviour that is associated with an imbalance of power.

Previous studies among children in kindergarten in Switzerland and the U.K. observed fairly similar patterns of teacher reported bullying and victimization as we did (e.g. bully-victims: 11% and 13%) [18,20]. However, research among young children in the U.S.A. indicated parent reported victimization rates of 23-27% [16,17]. These percentages are substantially higher than we observed, even when keeping in mind that victimized children in our study were found in two categories, i.e. the victims and the bully-victims. Differences in prevalence could be due to dissimilarities in the definition of victimization, but they might also be explained by the use of other informants, since teachers rate in a different context and with different references than parents [17,18]. On the other hand, a recent study indicated that the prevalence of victimization as reported by teachers or parents was fairly similar [44]. Another explanation comes from cross-national studies in older children and adolescents indicating that bullying and victimization rates are slightly higher in the USA than in the Netherlands [16,17,26,29].

### Socioeconomic disparities in bullying and victimization

The present study showed a strong socioeconomic gradient for different types of bullying and victimization with particularly marked differences in physical, verbal and relational bullying and victimization. Likewise, a strong association between family SES and involvement in bullying was shown: single parenthood, a young age and low educational level of parents were independently associated with the risk of children being bullies or bully-victims, which is in line with few previous studies in older children [13,28,29]. In contrast, being a victim was predicted by only a few indicators of family SES: only low maternal and paternal education was associated with a significant, nearly two-fold increased risk of victimization. Previous studies also found an educational gradient in victim status [19,24], but associations with other family SES indicators like single parenthood and parental occupation have been reported as well [19,23,25,26]. Results are, however, difficult to compare because the victimized children in our study were found in the victim and bully-victim categories.

The influence of several family socioeconomic characteristics was independent of school neighbourhood SES. Conversely, although greater neighbourhood socioeconomic disadvantage was associated with an increased risk of being a bully or bully-victim, this effect was not independent of family SES. This is in contrast with our empirically based hypothesis that school neighbourhood SES might affect bullying behaviour through various characteristics of school neighbourhoods [30,31]. A possible explanation is that prior intervention efforts and extra attention of teachers in socially disadvantaged neighbourhoods has resulted in a decrease in bullying prevalence in these areas, whereby the association between school neighbourhood SES and bullying has disappeared. It might also be that school neighbourhoods become more important when children are somewhat older. Our findings are, however, consistent with epidemiological research on other outcomes than bullying and victimization, suggesting that the effects of individual level SES might be stronger than neighbourhood SES effects [45].

Low socioeconomic background of families might have influenced children’s involvement in bullying and victimization in several ways. Parental educational level reflects intellectual resources, general and specific knowledge, norms and values, literacy, and problem solving skills [33,46], all aspects that could be related to child raising behaviour and, consequently, to children’s development of social skills and coping strategies. Additionally, it has been shown that children of low-educated parents watch more television than children of high-educated parents [47,48]. Possibly, exposure to violent television programs might stimulate bullying and peer aggression [49]. The association between single parenthood and the risk of children being a bully or bully-victim could be explained by less time for parent-children interaction. This could result in reduced parental control of children’s behaviour and limited time for parents to talk about the problems a child encounters in daily life, such as difficulties in peer relations. Alternatively, the effect of single parenthood could be accounted for by the stress inherent to a situation of broken families. Stress and parental well-being are known to have adverse influences on children’s behaviors in multiple ways [50]. Regarding employment status, we showed that children of whom both parents are unemployed were more likely to be a bully or bully-victim. This effect was explained by other SES indicators suggesting that parental unemployment is associated with children’s bullying behaviour through its relation with low educational level, single parenthood, and disadvantaged school neighbourhoods.

### Strengths and limitations

The present study was strengthened by its population-based design, large sample size, and the use of several socioeconomic indicators to conceptualize the multiple dimensions of SES [46,51]. Moreover, the multilevel models accounted for intra-class correlation arising from the fact that teachers reported bullying behaviour for all children in their classroom, and that children within the same class are more alike than children from different classes [31]. Limitations of this study include the use of a single informant of bullying and victimization. In principle, a teacher’s bias against children of lower socioeconomic backgrounds can affect ratings [52]. Multiple informants could also generate more accurate data on less overt bullying behaviours such as relational bullying [53]. Moreover, although we aimed to reduce teacher’s subjective opinions by providing examples and concrete descriptions of the different bulling and victimization types, the degree of agreement between teachers’ ratings is not known, as we did not assess inter-rater reliability. Furthermore, although bullying is a persistent process, a one-time measurement may coincide with some uncertainty due to changes in children’s behaviour and class composition over time. Another limitation of our study was that the non-response analyses indicated that the lack of information on SES was not completely random. Finally, we lacked possibilities to examine mechanisms explaining the association between SES and bullying behaviour at schools. Future studies should investigate the role of family and school influences, such as norms and values, and prevalence of vandalism.

### Implications

Our population-based study assessed prevalence of bullying and victimization among children in the first grades of elementary schools. This provides scholars and public health practitioners information on the prevalence of an important social behaviour that is a risk factor for later behavioural and emotional problems [2-8]. Considering the incessant nature of bullying and reports showing that by middle school both bully and victim roles are rather stable [54], the high prevalence of bullying and victimization shown in this study suggests the need of prevention and intervention programs at the start of elementary school. Our findings provide insight into which forms of bullying are common at this age, which is essential for tailored-made interventions targeting the most prevailing forms of bullying behaviour. Physical and verbal bullying was widespread; these overt behaviours can easily be recognized and are a possible target of intervention by school teachers. However, relational bullying was also a common behaviour that can be missed more easily. Therefore, it is important that teachers in early elementary school are made aware that relational bullying is a common behaviour in their class room, especially among girls. We also showed that children of families with a low socioeconomic background have a particularly high risk of involvement in bullying. The socioeconomic inequalities were not restricted to a specific type of bullying behaviour but were found in all forms of bullying and victimization. These findings should be taken into account in the development of bullying prevention or intervention programs as targeted programs may be more effective when actions are directed at the most prevailing forms of bullying and at the susceptible group of children. It might be worthwhile to teach children with a low socioeconomic background certain social skills and strategies to cope with peer problems and bullying situations. Possibly, children from families with a low SES do not learn such skills from their parents. The effectiveness of such intervention strategies and of general bullying interventions among young children in early elementary school should be monitored in future research.

## Conclusions

From previous research, it is known that bullying and victimization are widespread phenomena in secondary school and higher grades of elementary school. The present study adds to this literature by demonstrating that bullying behaviour is already a common problem in early elementary school. Children from socioeconomically disadvantaged families have an increased risk of being involved in bullying, especially as a bully or bully-victim. Our findings suggest the need of timely bullying preventions and interventions that should already be implemented at the start of elementary school. These programs should have a special focus on at-risk children of families with a low socioeconomic background. Future studies are necessary to evaluate the effectiveness of such programs.

## Abbreviations

SES: Socioeconomic status; OR: Odds ratio; FIML: Full information maximum likelihood.

## Competing interests

The authors declare that they have no competing interests.

## Authors’ contributions

PWJ conceptualized the study, performed statistical analyses and drafted the manuscript. MV, ADB and CM made substantial contributions to the acquisition and interpretation of the data. JE contributed to the design of the study and supervised the statistical analyses. RV, FCV and WJ made substantial contributions to the conception and design of the study. WJ was also involved in the interpretation of the data. HT made a substantial contribution to the conception and design of the study, interpretation of the data and supervised the drafting of the manuscript. All authors critically revised the manuscript and approved the final version of the manuscript.

## Pre-publication history

The pre-publication history for this paper can be accessed here:

http://www.biomedcentral.com/1471-2458/12/494/prepub

## Supplementary Material

Additional file 1: Table S1 Prevalence of victimization and bullying by educational level of the mother. Click here for file
