# Supplementary material for: Prevalence of bullying and victimization among children in early elementary school: Do family and school neighbourhood socioeconomic status matter?
Source: BMC Public Health. 2012 Jul 2;12:494. doi: 10.1186/1471-2458-12-494 (PMC3575320; doi:10.1186/1471-2458-12-494)
Supplement: Additional file 1: Table S1 — Prevalence of victimization and bullying by educational level of the mother. [file 1471-2458-12-494-S1.docx]

*Supplementary table 1. Prevalence of victimization and bullying by educational level of the mother*

|  |  | Percentage based on past 3 months | | | χ^2^ –test for overall difference |
| --- | --- | --- | --- | --- | --- |
| **Items** | **Educational level** | Never^#^ | Monthly | Weekly^§^ |  |
| **Victimization** |  |  |  |  |  |
| Physical | Higher academic  Higher vocational  Intermediate vocational  Lower vocational Primary education | 95.9  93.3  92.5  89.7  89.6 | 3.5  6.1  6.5  8.8  8.7 | 0.6  0.6  1.1  1.5  1.7 | <0.001 |
|  |  |  |  |  |  |
| Verbal | Higher academic  Higher vocational  Intermediate vocational  Lower vocational Primary education | 94.0  92.1  89.7  88.1  87.7 | 5.0  7.1  8.9  10.4  10.4 | 0.9  0.8  1.3  1.4  1.9 | 0.001 |
|  |  |  |  |  |  |
| Relational | Higher academic  Higher vocational  Intermediate vocational  Lower vocational Primary education | 94.3  92.8  92.2  90.2  85.5 | 4.9  6.7  6.5  8.5  13.0 | 0.8  0.5  1.3  1.3  1.4 | <0.001 |
|  |  |  |  |  |  |
| Material | Higher academic  Higher vocational  Intermediate vocational  Lower vocational Primary education | 99.7  99.4  99.5  99.2  97.8 | 0.2  0.6  0.5  0.8  1.5 | 0.2  0  0  0  0.7 | <0.001 |

*Supplementary table continued.*

|  |  | Percentage based on past 3 months | | | χ^2^ –test for overall difference |
| --- | --- | --- | --- | --- | --- |
| **Items** | **Educational level** | Never^#^ | Monthly | Weekly^§^ |  |
| **Bullying** |  |  |  |  |  |
| Physical | Higher academic  Higher vocational  Intermediate vocational  Lower vocational Primary education | 91.0  88.2  84.9  81.3  79.2 | 5.5  9.3  11.1  12.8  13.3 | 3.5  2.5  4.0  5.8  7.5 | <0.001 |
|  |  |  |  |  |  |
| Verbal | Higher academic  Higher vocational  Intermediate vocational  Lower vocational Primary education | 86.6  82.6  78.9  75.6  69.5 | 9.8  14.3  15.8  18.7  22.8 | 3.7  3.0  5.3  5.8  7.7 | <0.001 |
|  |  |  |  |  |  |
| Relational | Higher academic  Higher vocational  Intermediate vocational  Lower vocational Primary education | 89.4  87.4  84.0  82.6  77.2 | 9.3  11.9  13.3  14.2  16.9 | 1.2  0.8  2.7  3.2  5.8 | <0.001 |
|  |  |  |  |  |  |
| Material | Higher academic  Higher vocational  Intermediate vocational  Lower vocational Primary education | 99.1  97.9  97.0  97.2  94.0 | 0.5  2.1  2.5  2.3  4.8 | 0.5  0  0.5  0.5  1.2 | <0.001 |

Notes Table 1: ^#^ Never or less than once per month.

^§^ The categories of “One to two times per week” and “More than twice per week” were collapsed into the category “Weekly” due to very low prevalences.
